# 11β-Hydroxysteroid dehydrogenase type 1 contributes to the regulation of 7-oxysterol levels in the arterial wall through the inter-conversion of 7-ketocholesterol and 7β-hydroxycholesterol

**DOI:** 10.1016/j.biochi.2012.08.007

**Published:** 2013-03

**Authors:** Tijana Mitić, Ruth Andrew, Brian R. Walker, Patrick W.F. Hadoke

**Affiliations:** Endocrinology Unit, University/BHF Centre for Cardiovascular Science, College of Medicine and Veterinary Medicine, University of Edinburgh, The Queen's Medical Research Institute, Edinburgh EH16 4TJ, Scotland, UK

**Keywords:** 7-Oxysterol, Glucocorticoid, 11β-Hydroxysteroid dehydrogenase type 1, Aortic function, BHT, butylated hydroxytoluene, DMEM, Dulbecco's modified Eagle medium, EDTA, ethylenediaminetetraacetic acid, 11β-HSD1, 11β-hydroxysteroid dehydrogenase type 1, 11β-HSD2, 11β-hydroxysteroid dehydrogenase type 2, 7αOHC, 7α-hydroxycholesterol, 7βOHC, 7β-hydroxycholesterol, 7-KC, 7-ketocholesterol

## Abstract

The atherogenic 7-oxysterols, 7-ketocholesterol (7-KC) and 7β-hydroxycholesterol (7βOHC), can directly impair arterial function. Inter-conversion of 7-KC and 7βOHC has recently been shown as a novel role for the glucocorticoid-metabolizing enzyme 11β-hydroxysteroid dehydrogenase type 1 (11β-HSD1). Since this enzyme is expressed in vascular smooth muscle cells, we addressed the hypothesis that inter-conversion of 7-KC and 7βOHC by 11β-HSD1 may contribute to regulation of arterial function.

Incubation (4–24 h) of aortic rings with either 7-KC (25 μM) or 7βOHC (20 μM) had no effect on endothelium-dependent (acetylcholine) or -independent (sodium nitroprusside) relaxation. In contrast, exposure to 7-KC (but not to 7βOHC) attenuated noradrenaline-induced contraction (*E*_max_) after 4 h (0.78 ± 0.28 vs 0.40 ± 0.08 mN/mm; *p* < 0.05) and 24 h (2.28 ± 0.34 vs 1.56 ± 0.48 mN/mm; *p* < 0.05). Both 7-oxysterols were detected by GCMS in the aortic wall of chow-fed C57Bl6/J mice, with concentrations of 7-KC (1.41 ± 0.81 ng/mg) higher (*p* = 0.05) than 7βOHC (0.16 ± 0.06 ng/mg). In isolated mouse aortic rings 11β-HSD1 was shown to act as an oxo-reductase, inter-converting 7-KC and 7βOHC. This activity was lost in aorta from 11β-HSD1^−/−^ mice, which had low oxysterol levels. Renal homogenates from 11β-HSD1^−/−^ mice were used to confirm that the type 2 isozyme of 11β-HSD does not inter-convert 7-KC and 7βOHC.

These results demonstrate that 7-KC has greater effects than 7βOHC on vascular function, and that 11β-HSD1 can inter-convert 7-KC and 7βOHC in the arterial wall, contributing to the regulation of 7-oxysterol levels and potentially influencing vascular function. This mechanism may be important in the cardioprotective effects of 11β-HSD1 inhibitors.

## Highlights

► Metabolism of 7-oxysterols by 11β-HSD1 may influence arterial function. ► 11β-HSD1 inter-converted 7-ketocholesterol and 7β-hydroxycholesterol in mouse aorta. ► 7-ketocholesterol, but not 7β-hydroxycholesterol, altered aortic contraction. ► 11β-HSD1-mediated metabolism of 7-oxysterols may influence arterial contraction.

## 1 Introduction

Pro-atherogenic 7-oxysterols form a large component (40%) of oxidized LDL (oxLDL), of which 7-ketocholesterol (7-KC) contributes ∼30% [1]. 7-KC is toxic to cells in the vessel wall, and can impair arterial function *ex vivo* [2]. Indeed, 7-KC and its metabolite 7β-hydroxycholesterol (7βOHC) inhibited endothelium-dependent, acetylcholine-induced relaxation of rabbit aortic rings *in vitro* [2]. In human umbilical vein endothelial cells (HUVECs), 7βOHC and 7-KC reduced the histamine-activated release of nitric oxide [3]. This inhibition of endothelial function by 7-oxysterols appears to be independent of their cytotoxic effects [4], but its mechanism is unclear. Importantly, 7-KC and 7βOHC differ in their pro-atherogenic potential, with 7-KC implicated as the major pro-inflammatory and cytotoxic oxysterol [5]. However, any differences between the functional effects of 7-KC and 7βOHC in the vasculature have not been addressed.

The balance between 7-KC and 7βOHC in tissues may be actively modulated. Recently, a novel route of metabolism of 7-oxysterols has been described, involving the enzyme 11β-hydroxysteroid dehydrogenase (11β-HSD) type 1. The primary role of 11β-HSD1 is to catalyse the pre-receptor generation of glucocorticoids, allowing tissue-specific amplification of glucocorticoid receptor activation [6]. Inactivation of glucocorticoids is catalysed by the type 2 isozyme of 11β-HSD (11β-HSD2) [7]. It is becoming increasingly apparent that 7-oxysterols are alternative substrates for 11β-HSD1 [8,9], and inhibition of the enzyme can result in accumulation of 7-KC [10]. Since both isozymes of 11β-HSD are present in the arterial wall [11–14], where they are able to inter-convert glucocorticoids [15], it is conceivable that inter-conversion of 7-oxysterols by these enzymes has a role in modulating vascular function.

We used mice with targeted disruption of the 11β-HSD1 gene (*Hsd11b1*) to investigate the hypothesis that 11β-HSD1 metabolises 7-oxysterols in the arterial wall, thus influencing 7-KC- and 7βOHC-mediated modulation of arterial function.

## 2 Methods

### 2.1 Chemicals and stock solutions

All solvents were HPLC grade (Fisher, Hemel Hempstead, UK) and were prepared containing an anti-oxidant (0.01% w/v butylated hydroxytoluene (BHT)) to prevent oxidative degradation of the lipids [3]. Steroids and oxysterols were from Steraloids (Newport, Rhode Island, USA), derivatization reagents from Fluka (Buchs, Switzerland), tissue culture reagents from Lonza (Reading, UK) and other chemicals from Sigma-Aldrich (Poole, Dorset, UK). Deuterium-labelled internal standards for GCMS were obtained from CDN Isotopes (Qmx Laboratories, Essex, UK). Stock solutions (30 mg/ml in ethanol with 250 μg/ml BHT) of 7-KC, 7βOHC and 7αOHC (an optical isomer of 7βOHC) were freshly prepared as required. All steroids were prepared in 100% ethanol. Working solutions for tissue culture were prepared in standard Dulbecco's modified Eagle's medium (DMEM). Working solutions (25 μM 7-KC; 20 μM 7βOHC) for myography were prepared by diluting the appropriate stock solution in DMEM without l-Arginine (Arg) or phenol red, but containing 1% charcoal-stripped foetal calf serum. These were the maximum concentrations of 7-oxysterols that could be achieved without sample precipitation. The final concentration of vehicle (ethanol with 250 μg/ml BHT) was <0.2%.

### 2.2 Animals

Male mice (age 8–16 weeks) homozygous for disrupted alleles of 11β-HSD1 (*Hsd11b1*^−/−^) [16] or 11β-HSD2 (*Hsd11b2*^−/−^) [7], on a C57Bl6/J background [12] were bred in-house. Controls were age- and sex-matched C57Bl6/J mice bred in-house [7,16]. Mice were maintained on standard chow diet and tap water *ad libitum*, under a 16 h/8 h light/dark cycle at 21–24 °C. All procedures were performed under UK Home Office guidelines of humane care and [17,18] animals were culled by cervical dislocation at 10.00 h. Plasma (1 ml) was collected from 2–3 mice in EDTA-coated (1.6 mg/ml) vials (Sarstedt, Monovette) and separated by centrifugation (2000× *g*, 4 °C, 15 min), and an aliquot of BHT was added (50 μg/5 μl). Tissues were snap-frozen and stored at −80 °C until use. Aortae for functional investigation were removed from mice, placed in PBS (37 °C), cleaned of peri-adventitial fat and used for myography. Aortae for oxysterol analysis were processed as described below.

### 2.3 Functional effects of 7-oxysterols on isolated mouse aortic rings

Thoracic aortae were isolated from male C57Bl6/J mice (age 8–10 weeks, *n* = 12) and cut into four rings (2 mm in length). These were either used immediately for short-term (4 h), or incubated in a 24 well plate for extended (24 h), exposure to 7-oxysterols. For short-term exposures, aortic rings were mounted on intra-luminal wires in a small vessel wire myograph [19,20] containing DMEM without l-Arg (37 °C, continuously perfused with 95%O_2_: 5% CO_2_) [14]. After the vessels had been equilibrated at their optimum resting force they were contracted with KCl (125 mM, 3 times) to confirm viability and then incubated in the presence of: (1) 7-KC (25 μM in DMEM without l-Arg), (2) 7βOHC (20 μM in DMEM without l-Arg) or (3) vehicle alone (ethanol, with 50 μg/ml BHT in DMEM without l-Arg), for 4 h (2 rings/treatment/mouse). The incubating medium was replaced every 60 min. After 4 h cumulative concentration–response curves were obtained for 5-hydroxytryptamine (5-HT; 1 × 10^−9^–1 × 10^−4^ M) and noradrenaline (NA; 1 × 10^−9^–1 × 10^−4^ M). In addition, cumulative concentration–response curves were obtained for the vasodilators, acetylcholine (ACh; 1 × 10^−9^–1 × 10^−4^ M, endothelium-dependent) and sodium nitroprusside (SNP; 1 × 10^−9^–1 × 10^−4^ M, endothelium-independent), following contraction with a sub-maximal concentration of 5-HT (3 × 10^−7^–1 × 10^−6^ M). Contractile responses are expressed as force per unit length (mN/mm). Relaxations were expressed as a percentage of the contraction in response to the EC_80_ of 5-HT (% 5-HT).

For extended exposures [14], aortic rings were placed in a 24 well plate and immersed in 1 ml DMEM (without l-Arg) containing either 7-KC (25 μM), 7βOHC (20 μM), or vehicle (ethanol with 50 μg/ml BHT) and incubated overnight in a humidified incubator (37 °C; 5% CO_2_). These vessels were then mounted in a myograph and functional studies performed, as described above, in the continued presence of the appropriate 7-oxysterol or vehicle.

### 2.4 Determination of plasma and aortic levels of 7-oxysterols and cholesterol

Concentrations of cholesterol and 7-oxysterols in the plasma and aortae were quantified by GCMS. Aortae from *Hsd11b1*^−/−^ or C57Bl6/J mice were pooled from two animals, washed in PBS containing EDTA (0.5 mM), crushed under liquid nitrogen and homogenized. Protein concentration was determined using Bradford assay (Biorad, UK). Deuterium-labelled ([^2^H], d_7_) internal standards (IS) were added (50 μl) and lipids were extracted into chloroform/methanol (2:1, 8 ml) [21,22]. Samples were purified using Bond Elute Diol columns (100 mg, 1 ml; Varian, UK) [23] and hydrolysed following mild saponification [24,25]. Oxidized lipids and cholesterol were extracted from neutralized samples (0.35 ml, 20% acetic acid) into diethyl ether (4 ml, 0.01% BHT) and evaporated to dryness under argon. Total cholesterol and 7-oxysterol concentrations were measured by GCMS and corrected for aortic protein levels.

### 2.5 Metabolism of 7-oxysterols by 11β-HSD1 and 11β-HSD2 *in vitro*

#### 2.5.1 In the mouse aorta

Rings (2 mm long) of aortae from C57Bl/J6 and *Hsd11b1*^−/−^ mice (*n* = 8/group) were placed in a 24 well plate (1/well, in duplicate) and immersed in 1 ml DMEM (without l-Arg) containing 7-KC (25 μM), 7βOHC (20 μM), 7αOHC (20 μM) or vehicle (ethanol, with 50 μg/ml BHT). Samples were incubated overnight in a humidified incubator (37 °C; 5% CO_2_) then blotted dry on tissue paper and weighed to allow calculation of conversion velocity (pmol/mg/day). Medium was removed and deuterium-labelled internal standards (IS; [^2^H], d_7_-7-KC (40 ng), [^2^H], d_7_-7βOHC (10 ng) and [^2^H], d_7_-cholesterol (10 μg)) added in a single aliquot (50 μl). [^2^H], d_7_-7βOHC was used as an internal standard for quantitation of both 7αOHC and 7βOHC. Argon gas was flushed through all samples and oxysterols were extracted (8 ml, 100× *g*, 15 min) from media with a mixture of hexane:2-propanol (60:40) [26]. The organic phases were combined, evaporated under a stream of argon and residues dissolved in chloroform:methanol (2:1, 350 μl) before storing at −20 °C for analysis by GCMS. Results were subsequently corrected for aortic ring weight. In all assays appropriate positive controls were included, with aortic rings incubated with [^3^H], d_4_-corticosterone or [^3^H], d_4_-11-dehydrocorticosterone (30 nM) to verify the activity of 11β-HSD isozymes. Samples were processed for analysis as before [27].

#### 2.5.2 In kidney

Murine kidneys contain both isoforms of 11β-HSD. Homogenates of kidneys from *Hsd11b1*^−/−^ mice (which lack 11β-HSD1) were used as a source of murine 11β-HSD2, with kidneys from C57Bl6/J mice as controls. Kidneys were homogenized in phosphate buffer as detailed [10]. Homogenates (400 μg/ml) were incubated with 7-oxysterols (20 μM) and the appropriate cofactor (2 mM): NAD^+^ or NADP^+^ for dehydrogenase reactions; NADH or NADPH for reductase reactions. In all assays conversion of dexamethasone (Dex) and 11-dehydrodexamethasone (11-DHDex; 40 μM) was used as a positive control for confirmation of 11β-HSD isozyme activity [28].

### 2.6 Chromatographic analyses

#### 2.6.1 Analysis of 7-oxysterols by gas chromatography/mass spectrometry (GCMS)

7-Oxysterols and cholesterol were converted to trimethylsilyl ether derivatives using a pyridine:hexamethyldisilazan:trimethylchlorosilane mixture (350 μl, 3:2:1, v/v/v) [29,30]. The derivatized cholesterol metabolites were dissolved in 2% *N*,*O*-Bis(trimethylsilyl)trifluoroacetamide (BSTFA) in decane (80 μl) and eluted as follows: initial temperature 180 °C (1 min), increased by 35 °C/min until 270 °C was achieved (1 min) and then increased by 4 °C/min to 300 °C (12 min). The oven was then cooled by −10 °C/min to 250 °C (1 min). The injection temperature was 270 °C.

A capillary gas chromatograph (Trace GC, Thermo) was coupled to an ion-trap, Polaris Q (Thermo, Hemel Hempstead, UK) mass spectrometer (MS) and equipped with a BPX5 capillary column (25 m, 0.32 mm internal diameter and 0.25 μm film thickness; SGE, Alva, UK) and operated in SIM mode with electron impact (70 eV), ion source, transfer line and interface temperatures of 200 °C, 220 °C and 250 °C respectively. Derivatives of 7-oxysterols and cholesterol were quantified by monitoring the following ions (*m*/*z*): 7-KC (472, 16.5 min), 7α/βOH (456, 12.3 & 14.1 min), d_7_-7-KC (479, 16.35 min), d_7_-7βOHC (463, 13.8 min) and d_7_-cholesterol (375, 12.9 min). Limits of detection were assigned as 3:1 signal to noise ratio. Compounds were quantified by the ratio of area under peak of interest to area under peak of internal standard against a standard curve.

#### 2.6.2 Quantitation of steroids by high pressure liquid chromatography

Radio-labelled glucocorticoids were separated by reverse phase HPLC (Symmetry C8 column maintained at 35 °C; column length, 15 cm, internal diameter 4.6 mm, pore size 5 μm, Waters, Edinburgh, UK) and quantified by on-line liquid scintillation counting (2 ml/min; GoldFlow, Meridian, Surrey, UK). Total run time was 35 min (elution times of epi-cortisol, 11-dehydrocorticosterone and corticosterone were typically 12 min, 21 min and 31 min, respectively, with mobile phase of water:acetonitrile:methanol (60:15:25) at 1 ml/min). Dex and 11-DHDex were separated using a mobile phase of water:acetonitrile:methanol (55:20:25) at 1 ml/min with typical retention times for epi-cortisol (10 min), 11-DHDex (12 min) and Dex (16 min). UV detection of all steroids was achieved at 240 nm and epi-cortisol was used as an internal standard. Steroids were quantified by the ratio of area under peak of interest to area under peak of internal standard against a standard curve.

### 2.7 Statistical analysis

All data are mean ± standard error of the mean (SEM) where *n* indicates the number of different animals. Values were compared using unpaired Student's *t*-tests or 1-way ANOVA with Dunnett's multiple comparison post-tests, as appropriate. All analyses were performed using Graph Pad Prism v5.0 (GraphPad Software Inc. San Diego, USA). Statistical significance was assumed when *p* < 0.05.

## 3 Results

### 3.1 7-KC, but not 7βOHC, alters vascular function *in vitro*

**Fig. 1:**
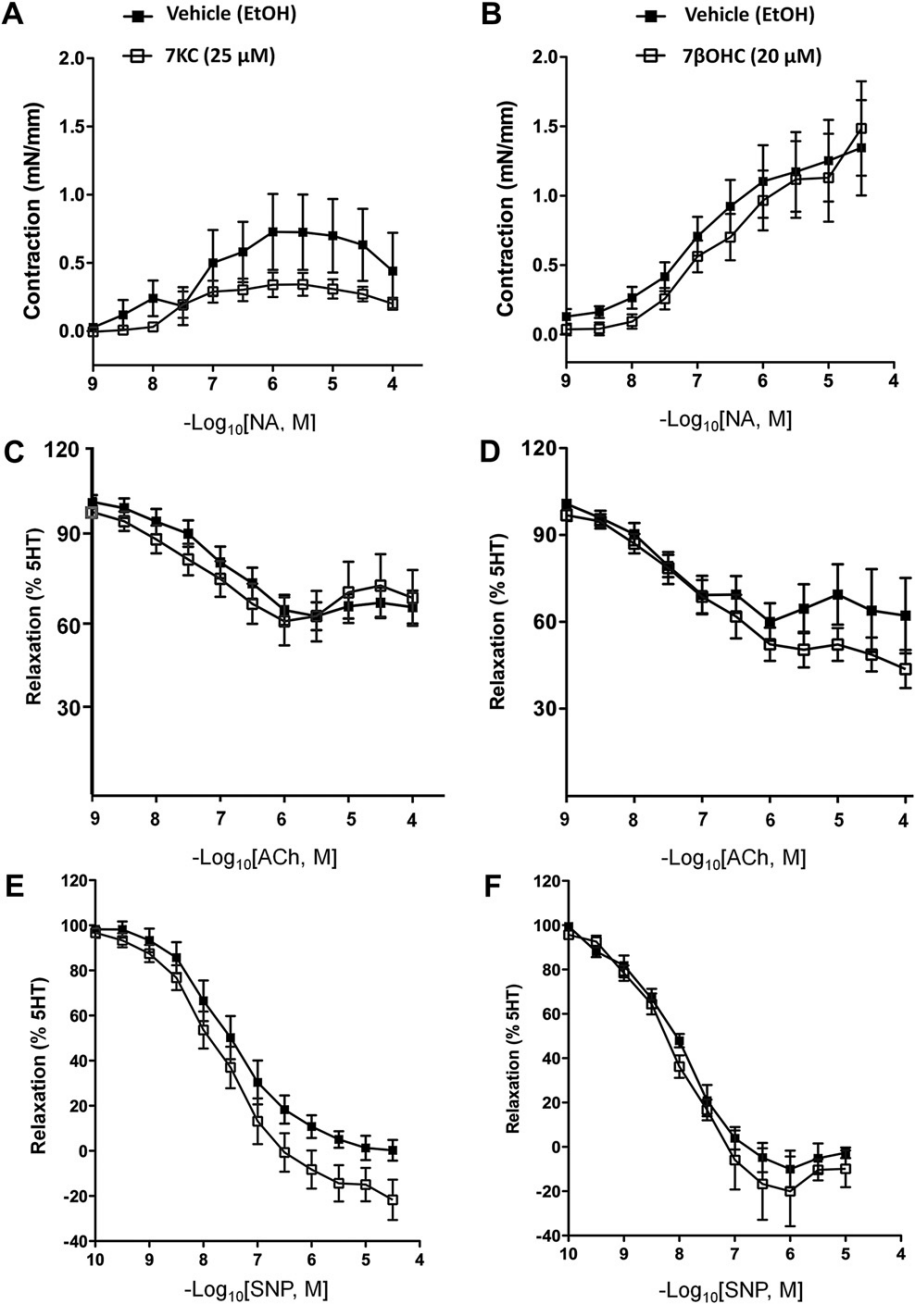
Short-term (4 h) exposure to 7-ketocholesterol induces agonist-specific functional changes in isolated mouse aorta. Endothelium-intact aortic rings from C57Bl6/J mice were incubated (4 h) with 7-ketocholesterol (7-KC, 25 μM open squares) or 7β-hydroxycholesterol (7βOHC, 20 μM, open squares) and compared with vehicle (ethanol containing 50 μg/ml butylated hydroxytoluene)-treated control (filled squares). Incubation with 7-KC (A), but not 7βOHC (B), produced a small reduction of noradrenaline (NA)-mediated contraction (*p* = 0.04). Incubations had no effect on acetylcholine (ACh)-mediated relaxation (C, D) whereas 7-KC (E) (but not 7βOHC (F)), produced a trend towards increased sodium nitroprusside (SNP)-mediated relaxation (*p* = 0.054). Relaxations were expressed on a scale where the response to 5-HT represented 100% and return to baseline was expressed as 0%. All points represent mean ± SEM, compared by 1-way ANOVA with Tukey's post hoc test, *n* = 6–8.

Short-term exposure (4 h) of aortae from C57Bl6/J mice to 7-KC (25 μM), but not 7βOHC (20 μM), produced a small reduction (*p* = 0.049) in NA-induced maximum contraction (*E*_max_), but had no effect on the sensitivity (*p*D_2_) of this response (Fig. 1A, B; Table 1A). 5-HT-mediated contraction was unaltered by exposure to either oxysterol (Table 1A). Pre-treatment of vessels with either 7-KC or 7βOHC did not alter endothelium-dependent relaxation to ACh (Fig. 1C, D). An apparent increase in maximal response to endothelium-independent, SNP-mediated vasorelaxation after incubation with either 7-KC (*p* = 0.05) or 7βOHC (*p* = 0.08) was of borderline statistical significance (Fig. 1E, F).

**Fig. 2:**
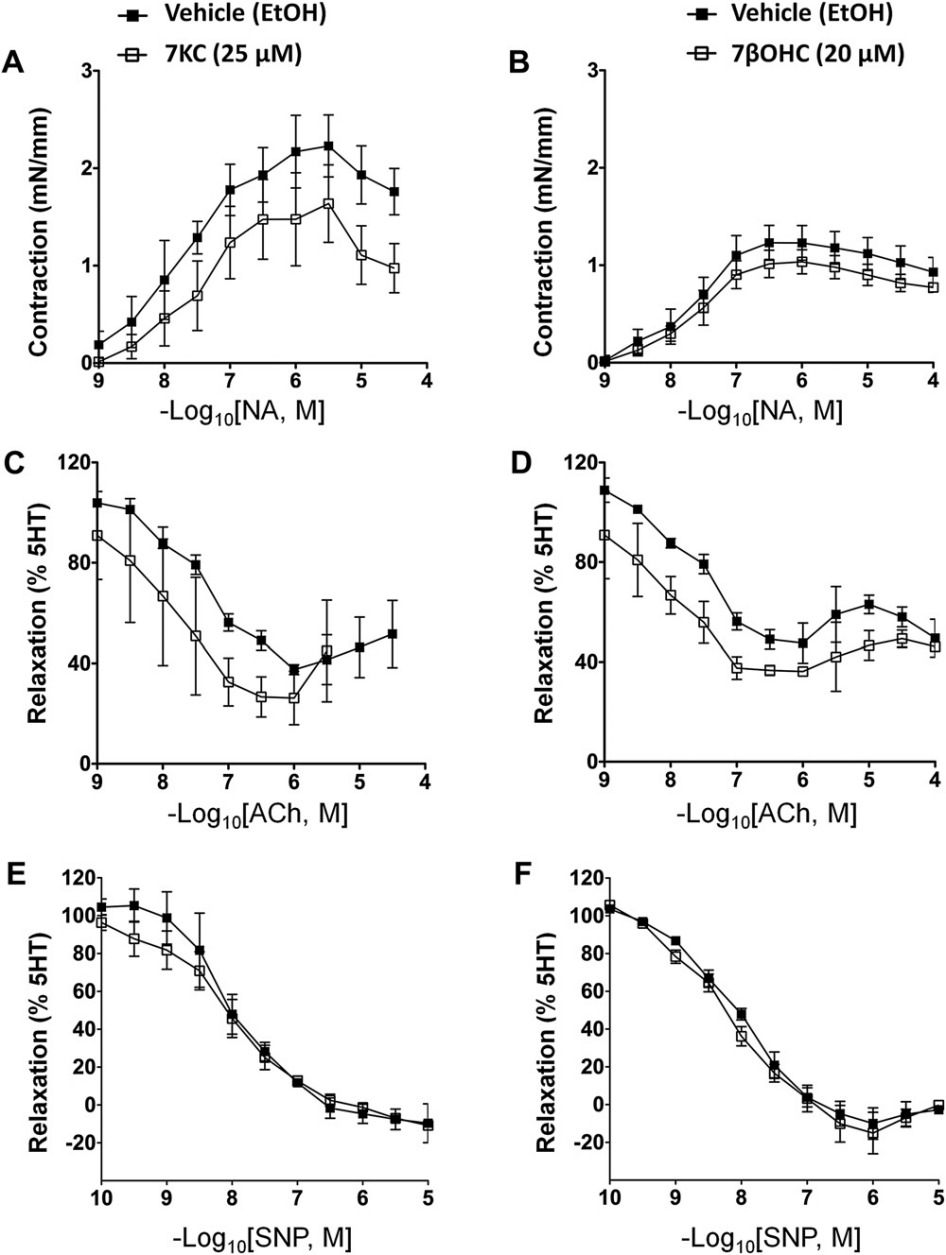
Long-term (24 h) exposure to 7-ketocholesterol induces agonist selective functional changes in isolated mouse aorta. Endothelium-intact aortic rings from C57Bl6/J mice were incubated (24 h) with 7-ketocholesterol (7-KC, 25 μM open squares) or 7β-hydroxycholesterol (7βOHC, 20 μM, open squares) and compared with vehicle (ethanol containing 50 μg/ml butylated hydroxytoluene)-treated control (filled squares). Incubation with 7-KC (A), but not 7βOHC (B), produced a small reduction of noradrenaline (NA)-mediated contraction (*p* = 0.05). Incubations had no effect on acetylcholine (ACh)-mediated (C, D) or sodium nitroprusside (SNP)-mediated (E, F) relaxation. All points represent mean ± SEM, compared by 1-way ANOVA with Tukey's post hoc test, *n* = 6–8.

Long-term (24 h; Fig. 2) exposure of aortae from C57Bl6/J mice to 7-KC (25 μM), but not 7βOHC (20 μM), produced a reduced maximum contraction (*E*_max_, *p* = 0.049), but no change in sensitivity (*p*D_2_) to NA (Fig. 2A, B; Table 1 B). Prolonged incubation with either 7-oxysterol had no effect on 5-HT-mediated contraction or endothelium-dependent (Fig. 2C, D) or -independent (Fig. 2E, F) relaxation (Table 1 B).

### 3.2 7-Oxysterols are present in the mouse aortic wall and altered by deletion of 11β-HSD1

7-KC (3.52 ± 2.85 nmol/g tissue) and 7βOHC (0.40 ± 0.15 nmol/g) were both detected in the mouse thoracic aortae with levels of 7-KC significantly higher than 7βOHC (*p* = 0.05; *n* = 12). In aortae from *Hsd11b1*^−/−^ mice, 7-KC was only present in levels above the limit of detection in 3 (of 8) samples and 7βOHC was also low (0.12 ± 0.02 nmol/mg). Plasma levels of 7-oxysterols were not different in *Hsd11b1*^−/−^ mice compared with C57Bl/6J mice (7-KC; 0.133 ± 0.016 versus 0.091 ± 0.022 μM; 7βOHC 0.024 ± 0.002 versus 0.023 ± 0.005 μM, respectively) although total plasma cholesterol was lower (0.45 ± 0.14 versus 1.03 ± 0.19 μM, *p* < 0.05).

### 3.3 7-Oxysterols are metabolized by 11β-HSD1 but not by 11β-HSD2

**Fig. 3:**
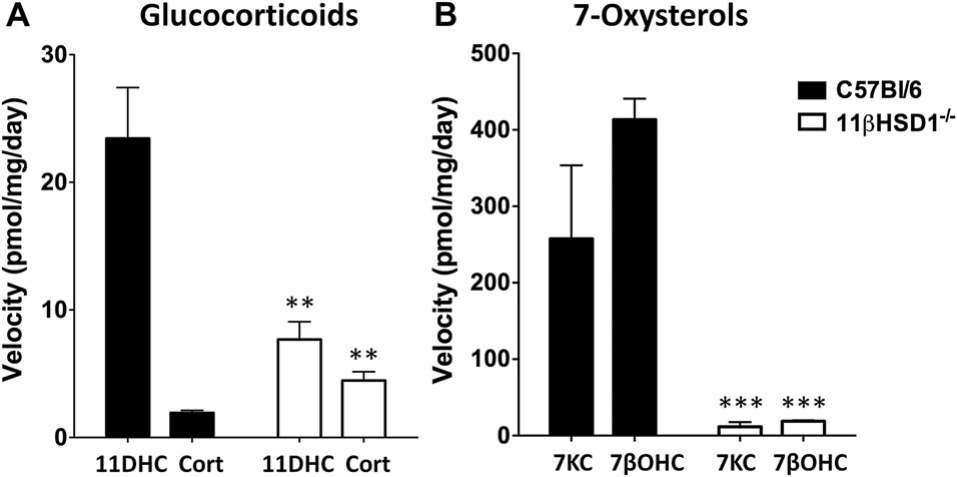
11β-HSD1 catalyses reduction of 7-Ketocholesterol (7-KC) and dehydrogenation of 7β-Hydroxycholesterol (7βOHC) in isolated mouse aorta. Incubation with mouse aortic rings (24 h; 37 °C; 5% CO_2_) resulted in (A) metabolism of glucocorticoids (*n* = 10); reduction of 11-dehydrocorticosterone (11DHC; 30 nM) to form corticosterone (Cort) was reduced, but not abolished, in aortae from 11β-HSD1^−/−^ mice. Low levels of dehydrogenation of Cort (30 nM; to form 11DHC) were detected in mouse aorta. Surprisingly this was slightly (but significantly) increased in the absence of 11β-HSD1. (B) Both 7-oxysterols (1 μM) were metabolized following exposure to mouse aortic rings (*n* = 6) but, in contrast to glucocorticoids, both dehydrogenation (conversion of 7βOHC to 7-KC) and reduction (7-KC to 7βOHC) reactions were virtually abolished in arteries lacking 11β-HSD1 (11β-HSD1^−/−^). Data are mean ± SEM, and were compared using unpaired Student's *t*-test, **p < 0.01, ***p < 0.001 vs velocity of the same reaction in tissues from C57Bl/6 mice.

As expected [15], glucocorticoids were inter-converted by incubation with intact mouse aortic rings. The velocity of reduction of 11-dehydrocorticosterone to corticosterone (Fig. 3A) proceeded considerably (∼10×) faster than the dehydrogenation of corticosterone to 11-dehydrocorticosterone. Reduction of 11-dehydrocorticosterone was attenuated in mice lacking 11β-HSD1, whereas deletion of this enzyme produced only a small (though significant) increase in the dehydrogenation of corticosterone (to 11-dehydrocorticosterone) (Fig. 3A). The oxysterols 7-KC and 7βOHC were also inter-converted by incubation with intact mouse aortic rings. In contrast to glucocorticoids, however, the velocities of reduction of 7-KC (to 7βOHC) and of dehydrogenation of 7βOHC (to 7-KC) were similar following incubation with mouse aortic rings (Fig. 3B). Genetic disruption of *Hsd11b1* significantly reduced the velocity of conversion of both 7-KC and 7βOHC (Fig. 3B), with 96 ± 6% of added substrates being recovered. 7-KC was not inter-converted with 7αOHC in aortic rings (data not shown).

*Hsd11b1*^−/−^ mouse kidney homogenates (a rich source of 11β-HSD2; [31]) were used to determine whether 7-oxysterols are metabolised by this isozyme. As with the aortic rings, conversion of glucocorticoids was used as a positive control for activity of 11β-HSD2 [14,27]. As anticipated, glucocorticoids were metabolized by mouse renal homogenates with preferential generation of 11-DHDex from Dex (oxidation; not shown). In contrast, renal homogenates did not inter-convert any of the 7-oxysterols (7αOHC, 7βOHC or 7-KC). Unrecovered substrate was ∼3% or lower for each compound (7αOHC, 1.9 ± 0.7%; 7βOHC, 3.2 ± 0.3%; KC, 2.1 ± 0.3%).

## 4 Discussion

This study shows for the first time that 11β-HSD1, but not 11β-HSD2, catalyses the conversion of 7-oxysterols in the vascular wall. Previous work has shown that murine and human 11β-HSD1 converts 7-KC to 7βOHC in the liver and in cultured adipocytes [8,9]. We provide evidence that murine 11β-HSD1 reduces 7-KC to 7βOHC in the vessel wall but, furthermore, that it also oxidizes 7βOHC to 7-KC. Use of *Hsd11b1*^−/−^ mice confirmed that 11β-HSD1 was the sole enzyme responsible for metabolism of 7-KC and 7βOHC in the aortic wall and that deletion of 11β-HSD1 alters vascular 7-oxysterol concentrations. Functional investigations showed differential effects of 7-KC and 7βOHC on vascular function, suggesting that this 11β-HSD1-mediated inter-conversion of 7-oxysterols may influence 7-KC-mediated inhibition of arterial contraction.

7-KC and 7βOHC have both been shown previously to inhibit endothelium-dependent vasorelaxation [32], cause endothelial cell death, and induce production of radical oxygen species [17,33]. This is consistent with the ability of oxidized lipids to impair the endothelium-dependent relaxation of aortic segments from hyperlipidaemic mice [34]. The lack of impact of exposure to oxysterols on endothelium-dependent relaxation was surprising given the previous indications that both 7-KC and 7βOHC inhibit endothelial function [2,3,32] *ex vivo*. One possible explanation for lack of effect on vasorelaxation is the use of a low concentration of 7-oxysterol (20–25 μM) compared with previous studies (180–270 μM; [2,3,32]). The concentrations used for our investigations were the highest we could achieve without precipitation and are consistent with that used (25 μM) to show 7-oxysterol-mediated smooth muscle apoptosis *in vitro* [35]. Furthermore, a recent investigation using high concentrations of 7-KC (205 μM) found no effect of *ex vivo* incubation on ACh-mediated relaxation of mouse aorta [36].

Intriguingly at the concentrations used in this investigation, there was an inhibition of smooth muscle cell contraction by 7-KC that was not observed with 7βOHC. The mechanism involved is unclear but the effect was selective for noradrenaline, suggesting an impairment in the α_1_-adrenoceptor signalling pathway. Impaired contractility is consistent with 7-KC at this concentration having detrimental effects on vascular smooth muscle cells [35]. These results suggest, therefore, that the balance of 7-KC and 7βOHC may have functional and structural implications in the arterial wall.

The concentrations of 7-oxysterols in the vessels of C57Bl6/J mice are consistent with those reported previously in human plasma and vessels [1,24]. Since circulating 7-oxysterols can be sequestered by cells in the vessel wall [37], we assessed the potential of vascular 11β-HSD1 to inter-convert 7-oxysterols in this tissue. Plasma 7-oxysterol levels were not altered in *Hsd11b1*^−/−^ mice although total plasma cholesterol was substantially lower. Consistent with previous reports of reduced intra-vascular cholesterol accumulation with inhibition of 11β-HSD1 [38], we found lower levels of all 7-oxysterols in the aortae of *Hsd11b1*^−/−^ mice. It was, therefore, difficult to assess intra-vascular 7-KC:7βOHC ratios, since 7-KC levels in particular were near to the detection limit, but the data suggest that 7-KC levels are disproportionately reduced in *Hsd11b1*^−/−^ mice, consistent with the enzyme acting predominantly as an oxidase (converting 7βOHC to 7-KC) *in vivo*.

The *ex vivo* incubation of aortic rings described here has not previously been used to assess inter-conversion of 7-oxysterols. This approach confirmed that the stability of 7-oxysterols can be preserved during incubation, as both 7-KC and 7βOHC were successfully recovered from DMEM. It had been postulated that 7-oxysterols may be taken up by the vessels during incubation but the percentage recovery of 7-oxysterols from reaction mixtures did not support this. Preparation of concentrated stock solutions of the 7-oxyserols proved unexpectedly difficult, despite using published methodology [3], with oxysterols precipitating at high concentrations. Based on our own experiences and advice from other groups 7-oxysterol solutions were prepared in DMEM containing FCS containing an antioxidant (BHT; to prevent oxidative degradation of the lipids [3]). It is unlikely that BHT would have a detrimental effect on vascular function as it did not alter histamine-induced NO production in cultured HUVECs [32].

*Ex vivo* assays clearly demonstrated that incubation of 7-oxysterols with mouse aortic rings results in the conversion of 7βOHC to 7-KC and 7-KC to 7βOHC, but not inter-conversion of 7αOHC and 7-KC. This is consistent with results generated in rats [9,10] and humans [39] but contrasts with the demonstration that 11β-HSD1 in hamsters can inter-convert 7αOHC and 7-KC [40]. The ability of 11β-HSD1 to inter-convert 7-oxysterols explains why carbenoxolone, a non-selective 11β-HSD inhibitor, attenuates 7-oxysterol metabolism in rat hepatic microsomes [10]. Interestingly, in contrast to the predominant reductase direction (11-dehydrocorticosterone to corticosterone) shown for metabolism of glucocorticoids, murine vascular 11β-HSD1 showed similar activity as both reductase (7βOHC to 7-KC) and dehydrogenase (7-KC to 7βOHC) for inter-conversion of oxysterols, consistent with previous reports in liver [9,39]. Under these assay conditions, the reaction velocity for inter-conversion of oxysterols was considerably (approximately 10-fold) higher than for reduction of 11-dehydrocorticosterone. This contrasts with the demonstration of similar reaction velocities observed in other studies [9,40] and is likely to be a consequence of study design as substrate concentrations were higher (∼800×) for the oxysterols than for the glucocorticoids.

Residual metabolism of glucocorticoids in aortae from *Hsd11b1*^−/−^ mice is consistent with vascular 11β-HSD2 expression [14,20]. Virtually no residual inter-conversion of 7βOHC and 7-KC was observed in aortae from mice lacking 11β-HSD1. Lack of 7-oxysterol metabolism by 11β-HSD2 was confirmed using kidney homogenates (since the kidney is rich in 11β-HSD2 [15]; using kidneys from *Hsd11b1*^−/−^ mice ensured that there was no interference from this isozyme). This finding is consistent with the previous attribution of 7-oxysterol metabolism solely to the action of 11β-HSD1 in hamster [40], rat [9,10], guinea pig [9,41] and human [39]. There was, however, a notable loss of substrate in the reaction mixtures; suggesting incomplete recovery of substrate, non-enzymatic degradation, or formation of alternative products [42]. There was no loss of substrate in blank samples (containing buffer but no tissue homogenate), confirming chemical stability of 7-oxysterols during the incubation.

Direct action on the cells of the arterial wall may not present the only mechanisms through which oxysterols can influence regulation of arterial function and structure. Previous work in our group [43] has indicated that the ability of oxysterols to act as substrates for 11β-HSD1 also makes them potential competitive inhibitors of glucocorticoid metabolism. This presents the possibility that endogenous 7-oxysterols contribute to regulation of 11β-HSD1-dependent glucocorticoid generation. Glucocorticoids can interact directly with the arterial wall to enhance vasoconstriction [44], impair endothelium-dependent relaxation [45], inhibit angiogenesis [27] and reduce vascular lesion formation. There is increasing evidence that these interactions are regulated by the activity of 11β-HSD1 [27,38]. However, it is notable that no systematic difference in vascular function has been observed in vessels from *Hsd11b1*^−/−^ mice [20], so whether alterations in either 7-oxysterol or glucocorticoids influences physiological vascular function remains uncertain. Perhaps interactions of oxysterols with 11β-HSD1 are more important in pathology. In healthy individuals, the maximum concentrations of 7-oxysterols [46,47] are lower than those in patients with atherosclerosis who may have levels of 7-oxysterols in the micromolar range [1]. It is plausible that inhibition of 11β-HSD1-mediated glucocorticoid generation in conditions of 7-oxysterol excess may have an indirect impact on arterial function and remodelling.

Metabolism of 7-oxysterols by 11β-HSD1 may also have implications for the development of new therapies. Selective 11β-HSD1 inhibition prevents atherosclerosis [38] and is being developed for treatment of cardiovascular risk factors [48], but the mechanisms responsible for this atheroprotective effect have not been demonstrated. It is conceivable that the beneficial effects of 11β-HSD1 inhibition are a consequence of prevention of 7-oxysterol inter-conversion as well as glucocorticoid metabolism.

## 5 Conclusions

11β-HSD1 influences 7-oxysterol concentrations within the arterial wall. By altering the balance of 7-ketocholesterol and 7β-hydroxycholesterol, 11β-HSD1 may modulate their specific effects on vascular function, especially in disease states in which oxysterol levels are increased.

## Figures and Tables

**Table 1:** Exposure to 7-oxysterols caused an agonist-selective inhibition of contraction, but had no effect on relaxation, of mouse aortic rings *in vitro*.

*A*) *Short* (4 h) *incubation*				
(i) 7-Ketocholesterol (7-KC)				
Agonist	*E*_max_ (mN/mm or % relaxation)		*p*D_2_/−logIC_50_	
Vehicle	7-KC	Vehicle	7-KC
NA	0.79 ± 0.28	**0.40** ± 0.08*	6.70 ± 0.34	7.92 ± 0.48
5-HT	3.01 ± 0.37	2.47 ± 0.26	6.36 ± 0.08	6.55 ± 0.06
ACh	46.3 ± 5.3	47.1 ± 7.0	6.32 ± 0.09	6.48 ± 0.09
SNP	104.8 ± 3.8	122.0 ± 8.3	7.53 ± 0.22	7.56 ± 0.12
(ii) 7β-Hydroxycholesterol (7βOHC)				
Agonist	*E*_max_ (mN/mm or % relaxation)		*p*D_2_/−logIC_50_	
Vehicle	7βOHC	Vehicle	7βOHC
NA	1.60 ± 0.36	1.80 ± 0.61	6.90 ± 0.17	6.80 ± 0.22
5-HT	2.80 ± 0.28	3.30 ± 0.32	6.40 ± 0.04	6.50 ± 0.05
ACh	47.7 ± 6.2	59.1 ± 4.8	7.30 ± 0.32	7.10 ± 0.16
SNP	116.7 ± 10.3	121.7 ± 12.0	7.90 ± 0.11	8.20 ± 0.11

*B*) *Extended* (24 h) *Incubation*				
(i) 7-Ketocholesterol (7-KC)				
Agonist	*E*_max_(mN/mm or % relaxation)		*p*D_2_/−logIC_50_	
Vehicle	7-KC	Vehicle	7-KC
NA	2.28 ± 0.34	**1.56** ± 0.48*	7.79 ± 0.16	7.94 ± 0.33
5-HT	4.03 ± 0.24	3.63 ± 0.33	6.61 ± 0.05	6.60 ± 0.12
ACh	66.9 ± 4.5	75.4 ± 6.4	7.47 ± 0.13	7.35 ± 0.21
SNP	108.6 ± 5.0	106.0 ± 1.5	7.04 ± 0.17	6.95 ± 0.19
(ii) 7β-Hydroxycholesterol (7βOHC)				
Agonist	*E*_max_ (mN/mm or % relaxation)		*p*D_2_/−logIC_50_	
Vehicle	7βOHC	Vehicle	7βOHC
NA	1.21 ± 0.14	1.16 ± 0.12	6.55 ± 0.06	6.24 ± 0.03
5-HT	2.43 ± 0.14	2.88 ± 0.15	6.51 ± 0.15	6.90 ± 0.32
ACh	46.4 ± 5.6	60.3 ± 5.2	7.17 ± 0.15	6.97 ± 0.31
SNP	104.6 ± 3.46	109.5 ± 1.94	6.84 ± 0.16	6.72 ± 0.19

All values represent mean ± SEM, compared by unpaired Student's *t*-test (vehicle vs 7-oxysterol), *n* = 4–8, *p < 0.05. NA, noradrenaline; 5-HT, 5-hydroxytryptamine; ACh, acetylcholine; SNP, sodium nitroprusside.

Bold represents significant differences in the data.
